# C-fos upregulates P-glycoprotein, contributing to the development of multidrug resistance in HEp-2 laryngeal cancer cells with VCR-induced resistance

**DOI:** 10.1186/s11658-017-0067-8

**Published:** 2018-02-20

**Authors:** Guodong Li, Xiaoling Hu, Lu Sun, Xin Li, Jianfeng Li, Tongli Li, Xiaohui Zhang

**Affiliations:** 1 Department of Otorhinolaryngology, Shanxi Provincial People’s Hospital Affiliated to Shanxi Medical University, Taiyuan, Shanxi 030012 China; 2 Department of Pharmacology, Shanxi Medical University, Taiyuan, Shanxi China; 3 Artificial Livers Treatment Center, Beijing YouAn Hospital, Capital Medical University, Beijing, 100069 China

**Keywords:** C-fos, P-glycoprotein, Multidrug resistance, Laryngeal carcinoma

## Abstract

**Background:**

Laryngeal cancer tends to have a very poor prognosis due to the unsatisfactory efficacy of chemotherapy for this cancer. Multidrug resistance (MDR) is the main cause of chemotherapy failure. The proto-oncogene c-fos has been shown to be involved in the development of MDR in several tumor types, but few studies have evaluated the relationship between c-fos and MDR in laryngeal cancer. We investigated the role of c-fos in MDR development in laryngeal cancer cells (cell line: human epithelial type 2, HEp-2) using the chemotherapeutic vincristine (VCR).

**Methods:**

HEp-2/VCR drug resistance was established by selection against an increasing drug concentration gradient. The expressions of c-fos and multidrug resistance 1 (mdr1) were measured using qPCR and western blot. C-fos overexpression or knockdown was performed in various cells. The intracellular rhodamine-123 (Rh-123) accumulation assay was used to detect the transport capacity of P-glycoprotein (P-gp, which is encoded by the mdr1 gene).

**Results:**

HEp-2 cells with VCR-induced resistance (HEp-2/VCR cells) were not only resistant to VCR but also evolved cross-resistance to other chemotherapeutic drugs. The expressions of the c-fos and mdr1genes were significantly higher in the HEp-2/VCR cells than in control cells. C-fos overexpression in HEp-2 cells (c-fos WT) resulted in increased P-gp expression and increased the IC_50_ for 5-FU. C-fos knockdown in the HEp-2/VCR cells (c-fos shRNA) resulted in decreased P-gp expression and decreased IC_50_ for 5-FU. An intracellular Rh-123 accumulation assay showed that the mean intracellular fluorescence intensity (MFI) was lower in the HEp-2/VCR cells than in HEp-2 cells. C-fos WT cells also showed lower MFI. By contrast, c-fos shRNA cells exhibited a higher MFI than the control group.

**Conclusion:**

C-fos increased the expression of P-gp and mdr1 in the HEp-2/VCR cells, and enhanced the efflux function of the cells, thereby contributing to the development of MDR.

## Background

Laryngeal cancer is a malignancy that originates in the laryngeal mucosal epithelial tissue. It has become the sixth most common cancer in the world, accounting for 5.7–7.6% of systemic malignant tumors and 7.9–35% of malignant tumors in the otorhinolaryngeal region [1]. Laryngeal squamous cell carcinoma (LSCC) is the most common form of laryngeal cancer, accounting for 93–99% of all laryngeal cancers [2]. The pathogenesis of LSCC may involve smoking, alcohol consumption, air pollution, viral infection, activation of proto-oncogenes and inactivation of tumor suppressor genes [3–6].

Currently, comprehensive regimens are used to treat LSCC, with surgery as the main intervention and radiotherapy, chemotherapy and immunotherapy as adjuvant interventions. Chemotherapy is included in the treatment of LSCC to improve the local control rate, reduce tumor recurrence and metastasis, enhance the cancer survival rate after therapy, and improve the quality of life of patients. However, the efficacies of chemotherapy based on vincristine (VCR), methotrexate (MTX), cisplatin (DDP) and 5-fluorouracil (5-FU) are far from ideal.

The main reason for this chemotherapy failure is the development of multidrug resistance (MDR) in tumor cells during the course of treatment [7, 8]. In recent years, the role of the proto-oncogene c-fos in tumor MDR has received increasing attention [9, 10]. C-fos is an important transcription factor in eukaryotic cells, where it induces mRNA transcription and protein expression of downstream genes, and participates in the regulation of cell proliferation and apoptosis [11]. It is also correlated with the differentiation, proliferation, invasion and metastasis of tumor cells [9, 12, 13] and is closely related to the prognosis of cancer patients [14–17].

C-fos involvement in the development of MDR in some tumors, such as breast cancer and ovarian cancer, has been theorized and studied [9, 18]. However, there have been few studies on the relationship between c-fos and MDR in LSCC.

To clarify the correlation between c-fos and MDR in LSCC, we induced drug resistance in human epithelial type 2 (HEp-2) cells through exposure to an increasing drug concentration gradient of VCR. The resulting cell line, denoted here as HEp-2/VCR, was used to examine the expressions of c-fos and mdr1, and the effects of c-fos overexpression or knockdown on both expression and function of P-glycoprotein (P-gp).

## Methods

### Establishment of the HEp-2/VCR cell line

HEp-2 cells, which are the most common cell line used for laryngeal cancer studies [19–21], were used as parental cells. They were obtained from the Cell Bank of Type Culture Collection of the Chinese Academy of Sciences.VCR (Zhejiang Hisun Pharmaceutical Co., Ltd.) was used to induce drug resistance via selection against an increasing drug concentration gradient. HEp-2 cells were first selected with 0.02 μmol/l VCR. After steady cell growth and passaging, the concentration of the drug was increased gradually. After 12 months of selection, the cells were found to grow steadily in the presence of 0.96 μmol/l VCR. The 3-(4,5-dimethylthiazol-2-yl)-2,5-diphenyl tetrazolium bromide (MTT) assay yielded a half maximal inhibitory concentration (IC_50_) value of 1.8 μmol/l. The resulting drug-resistant cell line was named HEp-2/VCR.

### Cell culture

HEp-2 and HEp-2/VCR cells were cultured in Dulbecco’s modified Eagle medium/nutrient mixture F-12 (HyClone) containing 10% fetal bovine serum (FBS) at 37 °C in a 5% CO_2_ incubator. To sustain the drug resistance of HEp-2/VCR cells, 1 μmol/l VCR was maintained in the medium during cell culture at all times. The drug was removed from the culture medium 2 weeks prior to experiments.

### Real-time PCR

The cells were harvested and washed twice with phosphate-buffered saline (PBS). Total RNA was extracted using Trizol reagent. The extracted mRNA was reverse transcribed into cDNA, and then subjected to PCR amplification (Takara Co., Ltd). The reaction mix (25 μl) was: 12.5 μl of SYBR Premix Ex Taq (2×), 1 μl of primer (10 μM), 0.5 μl of ROX Reference Dye II (50×), 1 μl of cDNA and 10.5 μl of ddH_2_O. PCR was performed as: 95 °C for 10 min, 40 cycles of 95 °C for 15 s, and 60 °C for 1 min. Real-time PCR primers suitable for specific amplification of c-fos and glyceraldehyde 3-phosphate dehydrogenase (GAPDH) were designed using the Primer 5 software according to sequences recorded in GenBank, and were synthesized by Sangon Biotech Co., Ltd. The sequences of the primers are shown in Table 1.The expression level of the target gene was calculated using the ΔΔCT method. GAPDH was used as an internal reference.

**Table 1:** The primer sequences used for PCR

Name		Sequence
c-fos WT^a^	sense:	5′- CGGAATTCATGATGTTCTCGGGCTTCAACG −3′
	antisense:	5′-TTGCGGCCGCTCACAGGGCCAGCAGCGTG-3′
c-fos shRNA^b^		5′-CCGGGACACACCCTTACTCTCCAAACTCGAGTTTGGA GAGTAAGGGTGTGTCTTTTT-3′
c-fos	sense:	5′-TGTCTGTGGCTTCCCTTGAT-3′
	antisense:	5′-ATCAAAGGGCTCGGTCTTCA-3′
mdr1	sense:	5′-AGAGGGGATGGTCAGTGTTG-3′
	antisense:	5′-GCTATCGTGGTGGCAAACAA-3′
GAPDH	sense:	5′-CAGCCTCAAGATCATCAGCA-3′
	antisense:	5′-GTCTTCTGGGTGGCAGTGAT-3′

^a^vector name PLVX- EGFP(2A)-puro, cleavage sites BamH1 and Not1

^b^vector name PLKO.1-puro, cleavage sites BamH1 and ECOR1

### Western blot

The cells were harvested and homogenized in RIPA lysis buffer (Sigma-Aldrich). The concentrations of the protein samples were determined by the Bradford method. The proteins were separated by sodium dodecyl sulfate polyacrylamide gel electrophoresis (SDS-PAGE, 10% gel) and transferred to membranes. After blocking with 5% skim milk for 1 h, the membrane was incubated with anti-P-gp antibody (Abcam), anti-c-fos antibody (Abcam) and anti-actin antibody (Sigma-Aldrich) at 4 °C overnight. The next day, the membranes were washed three times with Tris-buffered Saline-Tween 20 (TBST), and incubated with the secondary antibodies (Proteintech Group) for 1 h. After washing with TBST, the target proteins were visualized and subjected to image analysis.

### Construction of c-fos knockdown HEp-2/VCR cells and c-fos overexpressing HEp-2 cells

A recombinant plasmid capable of expressing short hairpin RNA (shRNA) against the c-fos gene, PLKO.1-puro-shRNA-fos, was constructed and transfected into HEp-2/VCR cells. The resulting cells were named c-fos shRNA.

A plasmid capable of overexpressing c-fos, PLVX-EGFP(2A)-puro-fos, was constructed and transfected into HEp-2 cells. The resulting cells were named c-fos WT.

HEp-2/VCR orHEp-2 cells were routinely inoculated into six-well plates before transfection. When the cells reached 70–90% confluence, lipofectamine 2000-mediated transfection was performed, followed by culture for 24–48 h. The blank control group (untransfected cells), the negative control (NC, transfected with empty vector) group and the transfected group were established simultaneously. 48 h after transfection, puromycin (2 μg/ml) was added to select for stable cell lines. To verify the efficiency of c-fos knockdown and overexpression, RT-PCR and western blot were performed to examine the expression levels of c-fos mRNA and protein, respectively. The primers of c-fos overexpression and knockdown and the restriction sites are summarized in Table 1.

### MTT assay

The HEp-2 or HEp-2/VCR cells were divided into the three groups (blank control, NC and transfected). Five replica wells were set up for each group. All groups were cultured in the presence of various concentrations of 5-FU (30–600 μM) for 48 h. Subsequently, 20 μl of MTT (5 mg/ml) was added to each well of cells, and incubated in the dark for 4 h. Then, the supernatant was discarded and 200 μl of dimethyl sulfoxide was added to each well. The optical density of each well was measured at 490 nm using a microplate reader (BioTek).

### Intracellular rhodamine-123 (Rh-123) accumulation assay

Cells from all the groups were harvested in the logarithmic growth phase and incubated in medium containing 1 mg/ml Rh-123 (Sigma-Aldrich Corporation) for 60 min at 37 °C with shaking. The cells were then washed twice with PBS.

Flow cytometric analysis was performed using a FACS Calibur (BD Biosciences) to determine the mean fluorescence intensity (MFI) in the cells (ex. 488 nm/em. 530 nm). All the experiments were repeated three times to obtain the average MFI values for each group.

### Statistical analysis

All the data are shown as means ± SD. Comparison between all groups was conducted using one-way analysis of variance (ANOVA) and comparison between two groups was conducted using Student’s t-test. The data were analyzed using the GraphPad Prism 5.0 software (GraphPad Software Inc.). *P* values less than 0.05 were considered statistically significant.

## Results

### Drug resistance of HEp-2/VCR cells

We established a drug-resistant human laryngeal carcinoma cell line, named HEp-2/VCR, by selection against an increasing drug concentration gradient. The IC_50_ of VCR was increased from 0.04 ± 0.01 μmol/l in the normal HEp-2 cells to 1.7 ± 0.19 μmol/l in the HEp-2/VCR cells (Table 2). The 42.5-fold increase in IC_50_ indicates successful establishment of the drug-resistant HEp-2/VCR cell line.

**Table 2:** Comparison of the IC_50_ values for HEp-2 and HEp-2/VCR cells exposed to 4 chemotherapeutics

	IC_50_/(μmol/l)		
Anti-cancer drugs			Resistant fold
	HEp-2	HEp-2/VCR	
VCR	0.04 ± 0.01	1.7 ± 0.19	42.5
MTX	1.2 ± 0.35	8.3 ± 0.23	6.90
DDP	0.5 ± 0.25	1.9 ± 0.16	3.8
5-FU	61.1 ± 4.35	332 ± 5.21	5.44

Data are shown as the means ± SD

The IC_50_ values for other common chemotherapeutic drugs were also assessed (Table 2). HEp-2/VCR cells were respectively 6.90, 3.8 and 5.44 times as resistant as HEp-2 cells to MTX, DDP and 5-FU. The results indicate that HEp-2/VCR is a multidrug-resistant cell line.

### Expression of c-fos and mdr1 in HEp-2/VCR cells

**Fig. 1:**
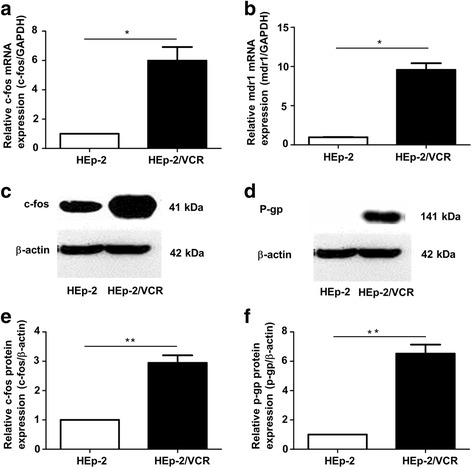
Differences in the expressions of c-fos and mdr1 (p-gp) in HEp-2 and HEp-2/VCR cells. **a** Expression of c-fos mRNA in HEp-2 and HEp-2/VCR cells. **b** Expression of mdr1 in HEp-2 and HEp-2/VCR cells. **c**, **d** Western blot analysis of the expression of c-fos and p-gp. **e**, **f** The statistical quantification analyses of c-fos and p-gp protein levels in HEp-2 and HEp-2/VCR cells. Data are shown as the means ±SD.*p < 0.05, **p < 0.01

Real-time PCR results showed that the expression of the proto-oncogene c-fos was low in HEp-2 cells, but increased 4.66-fold in the drug-resistant HEp-2/VCR cells (*p* < 0.05; Fig. 1a). The drug resistance gene mdr1 was expressed at low levels in HEp-2 cells but increased 9.57-fold in HEp-2/VCR cells (p < 0.05; Fig. 1b). The protein levels of c-fos and P-gp (which is encoded by mdr1) were also significantly elevated in HEp-2/VCR cells (Fig. 1c–f). This indicates a relationship between c-fos and mdr1 (P-gp).

### Expression of mdr1 and P-gp in HEp-2 cells overexpressing c-fos

**Fig. 2:**
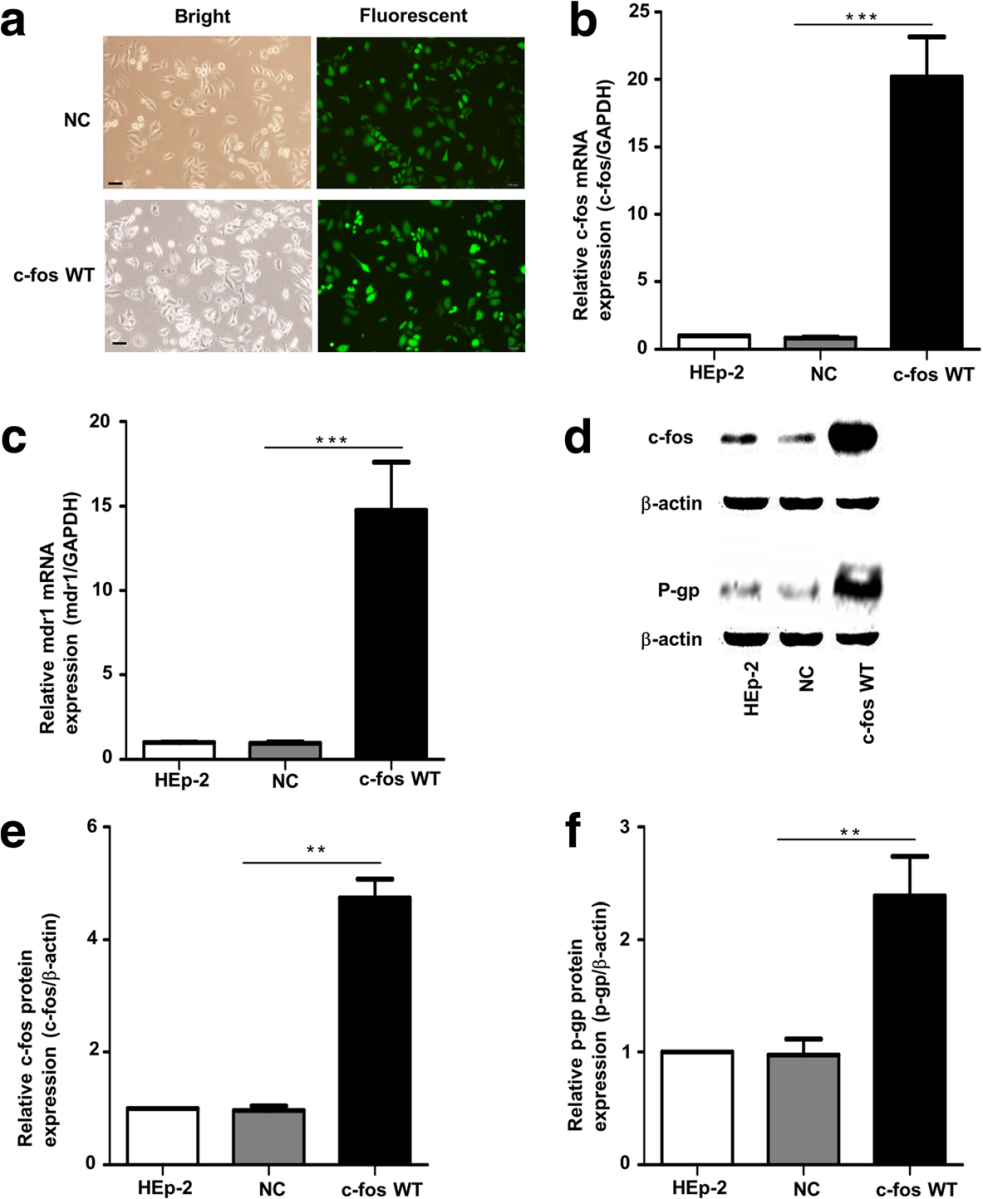
Expression of mdr1 and p-gp in HEp-2 cells after overexpression of c-fos. **a** Fluorescence microscopy examination of the efficiency of transfecting the c-fos overexpression plasmid into HEp-2 cells (named c-fos WT). The negative control (NC) was HEp-2 cells transfected with an empty vector. The scale bar represents 100 μm. **b**, **c** Real-time PCR results showing the expression of c-fos and mdr1 in HEp-2 cells after the overexpression of c-fos. **d** Western blot results showing the expression level of c-fos and p-gp in HEp-2 cells after the overexpression of c-fos. **e**, **f** The statistical quantification analyses of c-fos and p-gp protein levels in c-fos overexpression HEp-2cells.Data are shown as the means ±SD. **p < 0.01, ***p < 0.001

To confirm the correlation between c-fos and the drug resistance gene mdr1 and its corresponding protein P-gp, we overexpressed c-fos in HEp-2 cells and determined the expressions of mdr1 and P-gp. At 48 h after transfection, the transfection efficiency exceeded 90% (Fig. 2a), and the c-fos expression had significantly increased in the transfected HEp-2 cells, which were named the c-fos WT group (Fig. 2b, d, e). More importantly, both the expression of the mdr1 gene (Fig. 2c) and P-gp (Fig. 2d and f) was significantly higher in the c-fos WT group than in the HEp-2 cells and NC group.

### Expression of mdr1 and P-gp in HEp-2/VCR cells with c-fos knockdown

**Fig. 3:**
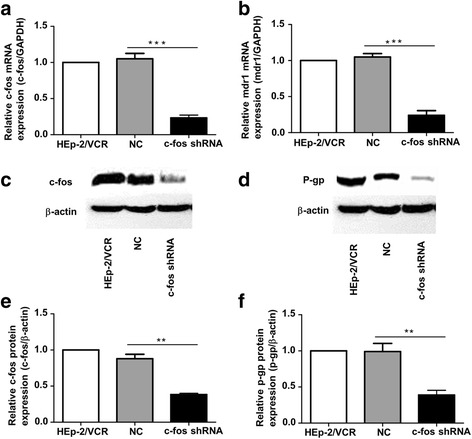
Expression of mdr1 and p-gp in HEp-2/VCR cells after c-fos knockdown. **a**, **b** Real-time PCR analysis of the expression of c-fos and mdr1 in various groups of cells. The HEp-2/VCR cells with c-fos knockdown were named the c-fos shRNA group, the negative control (NC) was HEp-2/VCR cells transfected with an empty vector. **c**, **d** Western blot assessment of the expression of c-fos and P-gp in various groups of cells. **e**, **f** The statistical quantification analyses of c-fos and p-gp protein levels in c-fos knockdown HEp-2/VCR cells. Data are shown as the means ±SD. **p < 0.01, ***p < 0.001

We further examined the expressions of mdr1 and P-gp in HEp-2/VCR cells after c-fos downregulation. HEp-2/VCR cells with c-fos knockdown, named the c-fos shRNA group, showed a very low expression of c-fos (Fig. 3a, c, e). Importantly, we found that the mdr1 expression in the c-fos shRNA group was reduced by more than 70%, compared to the NC group (*p* < 0.001; Fig. 3b). By contrast, there was no significant change in mdr1 expression between the NC group and HEp-2/VCR cells (Fig. 3b). A similar change was found in the protein level of P-gp (*p* < 0.01, Fig. 3d and f).

### Chemotherapeutic drug sensitivity of c-fos-overexpressing HEp-2cells and c-fos knockdown HEp-2/VCR cells

**Fig. 4:**
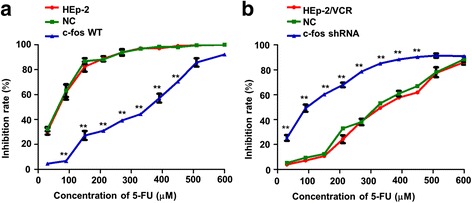
MTT assayof the changes in cell sensitivity to chemotherapeutic drugs after c-fos knockdown in HEp-2/VCR cells or c-fos overexpression in HEp-2 cells. All the groups of monoclonal cells were treated with various concentrations of 5-FU for 48 h. Concentration–effect curves showing the differences in the 5-FU IC_50_forvarious groups of cells. **a** The IC_50_ for 5-FU in the c-fos WT group was 268.5 ± 6.51 μmol/l, which was 4.86 times that of the NC group (55.2 ± 2.31 μmol/l; p < 0.01). **b** The IC_50_ of 5-FU had decreased by 75% in the c-fos shRNA group (80.2 ± 2.3 μmol/l) compared to the value for the NC group (309.2 ± 4.21 μmol/l; p < 0.01). Data are shown as the means ±SD. **p < 0.01

HEp-2, NC and c-fos WT cells were treated with various concentrations of 5-FU for 48 h and the corresponding changes in drug sensitivity were examined. The IC_50_ values for 5-FU were 55.2 ± 2.31 μmol/l in the NC group, and 268.5 ± 6.51 μmol/l in the c-fos WT group (4.86-foldincrease; *p* < 0.01; Fig. 4a). In the untransfected HEp-2 cells, the IC_50_ for 5-FU was 57.04 ± 3.21 μmol/l (no significant difference from the NC group; Fig. 4a). This indicates that overexpression of c-fos in HEp-2 cells led to significantly enhanced drug resistance.

Similarly, HEp-2/VCR, NC and c-fos shRNA cells were treated with various concentrations of 5-FU for 48 h. The IC_50_ values of 5-FU for the NC group and c-fos shRNA group were 309.2 ± 4.21 μmol/l and 80.2 ± 2.3 μmol/l, respectively (75%decrease, p < 0.01, Fig. 4b). By contrast, the IC_50_ for 5-FU in HEp-2/VCR cells was 312.9 ± 8.03 μmol/l, which was not significantly different to the NC group (Fig. 4b). This indicates that knockdown of c-fos in HEp-2/VCR cells led to significant reduction of drug resistance.

### C-fos changes the drug efflux function of P-gp in HEp-2 cells

**Fig. 5:**
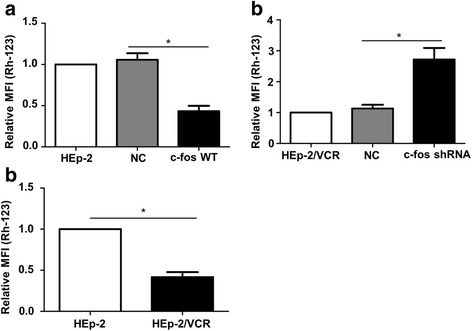
Effect of c-fos expression on the efflux function of P-gp. Cells were incubated with Rh-123 for 60 min, and then MFI (Rh-123) was determined using flow cytometric analysis. **a** The intracellular MFI was markedly lower in the c-fos WT group than in the NC group. **b** The intracellular MFI was significantly higher in the c-fos shRNA group than in the NC group. **c** The intracellular MFI (Rh-123) values for HEp-2/VCR cells and HEp-2 cells. Data are shown as the means ±SD. *p < 0.05

To investigate the role of c-fos in the development of MDR in HEp-2 cells, we examined the effect of c-fos on the efflux pump activity of P-gp. The MFI values for Rh-123 were compared between the groups, using P-gp as the substrate and Rh-123 as the fluorescent probe. As shown in Fig. 5, the MFI (Rh-123) values of HEp-2 and HEp-2/VCR cells were not significantly different from those of their corresponding NC groups. However, the intracellular MFI in the c-fos WT group was approximately 60% lower than that for the NC group (*p* < 0.05; Fig. 5a). The MFI in the c-fos shRNA group had increased 2.72-fold compared to the NC group (p < 0.05; Fig. 5b). In addition, we also found the efflux pump activity of P-gp is increased in HEp-2/VCR (p < 0.05; Fig. 5c).

## Discussion

The role of chemotherapy in the treatment of head and neck cancers was officially confirmed in the 1970s [19]. Preoperative induction chemotherapy is conducive to reducing the postoperative recurrence rate and improving the survival rate of patients. The administration of adjuvant chemotherapy after surgery or radiotherapy may inhibit or kill tumor micro-foci that cannot be distinguished by the naked eye during surgery or radiotherapy [20]. However, the efficacy of chemotherapy in the treatment of LSCC is currently not satisfactory [21].

The development of MDR may be the main cause of chemotherapy failure. For example, a two- to threefold increase in drug resistance in patients with advanced cancer will result in significantly reduced treatment efficacy. To date, the mechanisms underlying the development of MDR have not been completely elucidated [22].

MDR considerably restricts the success of clinical treatments for LSCC. Therefore, studying the mechanisms of drug resistance in LSCC is of great value for enhancing the efficacy of chemotherapy and guiding clinical decisions regarding medications.

In this study, we examined the differences in c-fos expression and cell sensitivity to chemotherapeutic drugs in two laryngeal cancer cell lines: drug-sensitive HEp-2 and drug resistant HEp-2 (HEp-2/VCR). Our results demonstrate that c-fos-mediated enhancement of the efflux function of P-gp in cells may be one of the mechanisms underlying the development of MDR in LSCC.

Drug resistance was induced via the culture of the VCR-sensitive parental cell line HEp-2 against an increasing drug concentration gradient. The resulting cell line, denoted HEp-2/VCR, was demonstrated to have MDR.

The proto-oncogene c-fos plays an important role in tumor cell proliferation, transformation and angiogenesis and tumor infiltration and metastasis [23]. It is reported that there is a correlation between c-fos and MDR in breast cancer and ovarian cancer [9, 18]. Some data also revealed that c-fos already plays a role in the early stage of the development of the drug-resistant phenotype in breast cancer. Knockdown of c-fos results in enhanced cell sensitivity to chemotherapeutic drugs, such as 5-FU and DDP.

Our study showed that the expression of c-fos was significantly higher in HEp-2/VCR than in HEp-2 cells. A relationship between c-fos and MDR was hypothesized for LSCC. To prove the hypothesis, we overexpressed c-fos in HEp-2 cells. Drug resistance was significantly enhanced. Knockdown of c-fos in HEp-2/VCR cells decreased drug resistance. This further confirmed the close relationship between c-fos and MDR, meaning that c-fos may be a target for enhancing the drug sensitivity of tumor cells to support chemotherapeutic treatment of LSCC.

P-gp is an adenosine triphosphate-dependent transmembrane efflux transporter that is widely distributed in tissues and organs throughout the body. It plays an important role in the absorption, distribution, metabolism and excretion of various drugs. As the coding gene of P-gp, mdr1 has a great impact on the susceptibility to many diseases and the pharmacokinetics, pharmacodynamics and therapeutic effects of certain drugs in vivo. It is reported that P-gp induces tumor cell resistance to a number of anti-tumor drugs, so mdr1 has become a molecular marker for the diagnosis of a variety of cancers and is a consideration when determining the prognosis of various cancers [24, 25].

Our previously published data show that c-fos downregulation could result in decreased P-gp expression and activity and enhanced apoptosis [9], suggesting that c-fos affects the expression of P-gp. Here, when c-fos was overexpressed in HEp-2 cells (c-fos WT), the expressions of mdr1 and p-gp protein increased accordingly. By contrast, c-fos knockdown in HEp-2/VCR cells (c-fos shRNA) resulted in decreased expressions of mdr1 and p-gp. It indicates that the expression of mdr1 and P-gp is closely related to c-fos, which is consistent with the results of our previous study [9].

Variations in P-gp affect drug efflux and transport, thus affecting the drug resistance of cells. In our study, the Rh-123 accumulation experiment mimicked the situation of a chemotherapeutic drug in the intracellular space. The intracellular MFI (Rh-123) in HEp-2/VCR was lower than that for HEp-2 cells. Overexpression of c-fos in HEp-2 cells also resulted in a reduced MFI, indicating that more Rh-123 was transported outside the cells through P-gp. However, knockdown of c-fos in HEp-2/VCR cells resulted in enhanced intracellular MFI, indicating that the transport capacity of P-gp was significantly reduced after P-gp downregulation, which led to the accumulation of a large amount of Rh-123 in the cells.

These results confirm our hypothesis that c-fos reduces the sensitivity of cells to chemotherapeutic drugs by increasing the expression of P-gp and enhancing the efflux function of cells. We conclude that mdr1 and P-gp constitute one of the mechanisms by which c-fos affects the development of MDR in tumor cells.

## Conclusions

Based on this evidence, c-fos can increase the expression of P-gp and mdr1 in laryngeal cancer cells and enhance the efflux function of the cells, thereby contributing to the development of MDR. However, understanding the complex mechanisms and signal transduction pathways that are believed to govern the role of c-fos in multidrug resistance of laryngeal cancer cells will require further study.

## Abbreviations

5-FU: 5-fluorouracil
ATP: Adenosine triphosphate
c-fos shRNA: c-fos knockdown in HEp-2/VCR cells
c-fos WT: c-fos overexpression in HEp-2 cells
DDP: Cisplatin
FBS: Fetal bovine serum
GAPDH: Glyceraldehyde 3-phosphate dehydrogenase
IC_50_: a half maximal inhibitory concentration
LSCC: Laryngeal squamous cell carcinoma
MDR: Multidrug resistance
mdr1: Multidrug resistance 1
MFI: Mean fluorescence intensity
MTT: 3-(4,5-dimethylthiazol-2-yl)-2,5-diphenyl tetrazolium bromide
MTX: Methotrexate
PBS: Phosphate-buffered saline
P-gp: P-glycoprotein
Rh-123: Intracellular rhodamine-123
shRNA: Short hairpin RNA
TBST: Tris-buffered saline-Tween 20
VCR: Vincristine
